# *Otx2* expression and implications for olfactory imprinting in the anemonefish, *Amphiprion percula*

**DOI:** 10.1242/bio.20135496

**Published:** 2013-07-17

**Authors:** Heather D. Veilleux, Lynne Van Herwerden, Nicholas J. Cole, Emily K. Don, Christian De Santis, Danielle L. Dixson, Amelia S. Wenger, Philip L. Munday

**Affiliations:** 1 School of Marine and Tropical Biology, James Cook University, Townsville QLD 4811, Australia; 2 Centre for Tropical Fisheries and Aquaculture, James Cook University, Townsville QLD 4811, Australia; 3 School of Medical Science and Bosch Institute, The University of Sydney, NSW 2006, Australia; 4 Australian School of Advanced Medicine, Macquarie University, Sydney, NSW 2109, Australia; 5 School of Biology, Georgia Institute of Technology, Atlanta, GA 30332, USA; 6 ARC Centre of Excellence for Coral Reef Studies, James Cook University, Townsville QLD 4811, Australia

**Keywords:** Olfaction, Coral reef fish, Habitat selection, Memory, Candidate gene, *In-situ* hybridisation

## Abstract

The *otx2* gene encodes a transcription factor (OTX2) essential in the formation of the brain and sensory systems. Specifically, OTX2-positive cells are associated with axons in the olfactory system of mice and *otx2* is upregulated in odour-exposed zebrafish, indicating a possible role in olfactory imprinting. In this study, *otx2* was used as a candidate gene to investigate the molecular mechanisms of olfactory imprinting to settlement cues in the coral reef anemonefish, *Amphiprion percula.* The *A. percula otx2* (*Ap-otx2*) gene was elucidated, validated, and its expression tested in settlement-stage *A. percula* by exposing them to behaviourally relevant olfactory settlement cues in the first 24 hours post-hatching, or daily throughout the larval phase. *In-situ* hybridisation revealed expression of *Ap-otx2* throughout the olfactory epithelium with increased transcript staining in odour-exposed settlement-stage larval fish compared to no-odour controls, in all scenarios. This suggests that *Ap-otx2* may be involved in olfactory imprinting to behaviourally relevant settlement odours in *A. percula*.

## Introduction

The *orthodenticle homeobox 2* (*otx2*) gene encodes a DNA-binding transcription factor (OTX2) involved in rostral head development, including the development of the olfactory, auditory and visual systems in a wide range of animals (Simeone et al., 1993; Matsuo et al., 1995; Kablar et al., 1996; Ueki et al., 1998). While first described in *Drosophila* (*otd*; Finkelstein et al., 1990; Wieschaus et al., 1992), orthologues were soon after described in mammals (murine *otx1* and *otx2*; Simeone et al., 1992). With a well-conserved homeodomain, *otx* orthologues are now known in a wide range of metazoans, from diploblastic cnidarians (Müller et al., 1999; Smith et al., 1999), to deuterostomes, such as echinoderms (Gan et al., 1995), ascidians (Wada et al., 1996), and a variety of vertebrates. *Otx* gene duplications are purported to have occurred in a gnathostome ancestor, and, subsequently, each paralogue has functionally diversified (Germot et al., 2001; Suda et al., 2009). While the paralogues *otx1* and *otx2* share a similar and synergistic function in patterning of the developing head (Suda et al., 1996), *otx5* and its mammalian orthologue, cone rod homeobox (*crx*; Plouhinec et al., 2003), are involved in the differentiation of retinal photoreceptors and circadian entrainment to daily light–dark cycles (Furukawa et al., 1999; Gamse et al., 2002).

In mice, the deformation or complete lack of olfactory epithelium in embryos with an inactivated copy of *otx2* (*otx2*^+/−^; Matsuo et al., 1995), combined with the early presence of OTX2 in the embryonic olfactory placode (Mallamaci et al., 1996), suggests that OTX2 plays an integral role in the olfactory system. In addition, cells expressing OTX2 are physically associated with olfactory axons and ensheath axon bundles arising from the olfactory epithelium and vomero-nasal organ and heading towards the telencephalon (Mallamaci et al., 1996). In adult zebrafish, exposure to an artificial odour, PEA (phenyl-ethyl-alcohol), during the first three weeks of life caused *otx2* (among other genes) to be upregulated 3.26 fold in the olfactory epithelium when compared to unexposed control fish (Harden et al., 2006). The odour-exposed fish had significantly more cells expressing *otx2* in the olfactory epithelium than control fish, starting from as early as 24 hours post fertilisation and then throughout development into adulthood. In contrast, cells expressing *otx2* in the midbrain were similar in both treatment and control fish, indicating the expression changes are specific to the olfactory epithelium. Harden et al. posit that these expressional changes play a role in olfactory memory due to epigenetic processes in the olfactory epithelium (Harden et al., 2006).

In many coral reef fish, olfaction plays a critical role in locating settlement habitat following a larval dispersal stage (Atema et al., 2002; Lecchini et al., 2005; Gerlach et al., 2007; Leis et al., 2011). For example, larvae of several different reef fish species prefer water from their settlement reef when exposed to waters from a variety of nearby reefs (Gerlach et al., 2007; Dixson et al., 2008; Dixson et al., 2011; Miller-Sims et al., 2011). This indicates that each reef has a distinct repertoire of odours and that fish have the ability to detect these different odours. Similarly, navigation to reefs and locations of appropriate settlement habitat appears to be mediated by olfactory cues in the orange anemonefish, *Amphiprion percula*. Despite the possibility of being carried away from their natal reef by currents, over sixty percent of *A. percula* settle on their natal reef after 10 to 13 days in the water column as larvae (Almany et al., 2007). While exact mechanisms that enable *A. percula* larvae to locate suitable settlement sites are unknown, Dixson et al. suggest that an olfactory response to chemical cues of island vegetation may play a role (Dixson et al., 2008). *A. percula* and its host anemones, *Heteractis magnifica* and *Stichodactyla gigantea*, are most abundant on coral reefs surrounding vegetated islands rather than on emergent reefs, and anemonefish larvae exhibit a strong behavioural preference for seawater containing olfactory cues from rainforest tree leaves (Dixson et al., 2008). Therefore, chemical cues released by island vegetation might assist larvae to navigate to reefs where suitable settlement habitat can be found.

Anemonefish recruits also respond to olfactory cues released from anemones, enabling them to locate a suitable host for settlement once they have navigated to reef habitat at the end of their pelagic larval stage (Arvedlund and Nielsen, 1996). Arvedlund et al. demonstrated that the specialist, *Amphiprion melanopus*, not only has an innate preference for its host anemone but that this preference is augmented by an imprinting mechanism (Arvedlund et al., 1999). As anemonefish spawn on substrate close to their host anemones, imprinting likely occurs when the anemone mucous comes into close contact with the eggs (Arvedlund et al., 2000a). As olfaction appears to be an important homing and habitat selection mechanism in anemonefish, these species provide a useful model group for testing the genetic mechanisms of olfactory memory formation in reef fishes (Hino et al., 2009).

As *otx2* is upregulated in odour-exposed zebrafish compared to controls, it is an ideal candidate gene to investigate olfactory imprinting in anemonefish. The aims of this study, therefore, were to: (1) elucidate the sequence of the *otx2* gene in the anemonefish, *A. percula* (*Ap-otx2*); (2) compare Ap-OTX2 to other OTX sequences to validate its identity and discriminate between it and other possible OTX paralogues; (3) determine if *Ap-otx2* is expressed in the olfactory area of *A. percula*; (4) test if there is variation in *Ap-otx2* transcript abundance in settlement cue exposed fish compared to controls; and (5) assess the utility of this gene for the future detection of olfactory imprinting in anemonefish.

## Materials and Methods

### Isolation of *Ap-otx2*

The *Danio rerio otx2* gene (*Dr-otx2*), EST accession number U14592, was used to design primers for amplification of a portion of the *Ap-otx2* gene corresponding to *Dr-otx2* exon four. *Dr-otx2* is located on chromosome 17 and has four exons (E1–E4; Ensembl database). Each *Danio rerio* exon was aligned using BioEdit Sequence Alignment Editor Software (Hall, 2007) to *otx2* sequences from five species of cichlid and *Takifugu rubripes* available from the GenBank Public Database (Altschul et al., 1997; accession numbers AB084643, DQ264396, AB084641, AB084641, AB084644, AY303542, NM_131251 respectively). Using conserved regions from these sequences, a pair of primers was designed for use in the Polymerase Chain Reaction (PCR) to amplify a 374 base pair (b.p.) region in the coding region of E4 (Table 1; *Ap-otx2*-E4 F and R). E1 and E2 were not selected for primer design as there is a high degree of sequence variability among species due to the untranslated region and the E3 sequence has high similarity to other *otx* paralogues, *otx1* and *otx5*.

**Table 1.**
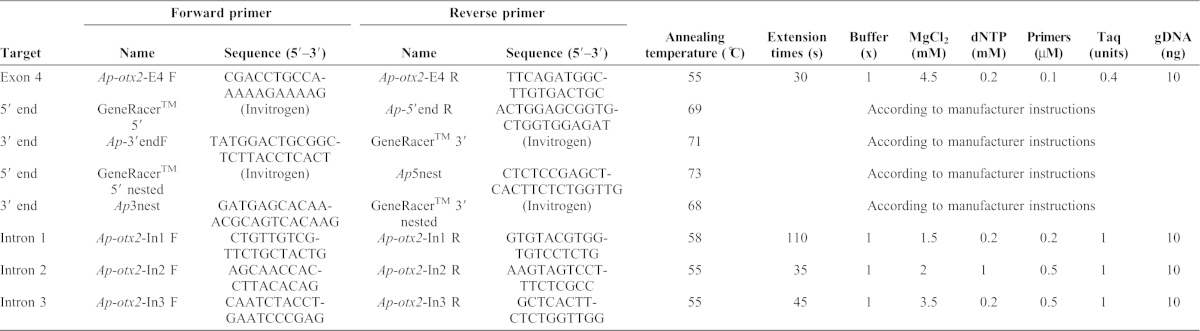
Primer sequences and polymerase chain reaction (PCR) conditions for amplification of the *Amphiprion percula otx2* sequence.

Fin clips were collected from four juvenile *A. percula* housed in 70-litre tanks in a 70,000-litre recirculating seawater system at the James Cook University's Marine Aquarium Facility. Total genomic DNA (gDNA) was extracted using a Chelex extraction protocol (Walsh et al., 1991). The *Ap-otx2*-E4 primers were used in a 20 µL PCR according to conditions listed in Table 1, using Qiagen reagents. Samples were amplified in a Peltier Thermal Cycler (Bio-Rad) with the following steps: pre-denaturation at 96°C for 2 min; 35 cycles of denaturation for 30 sec at 96°C, annealing at 55°C for 30 sec and extension at 72°C for 30 sec; followed by a 10 min final extension. PCR products were checked by separating on a 1.5% agarose gel, purified using the QIAquick PCR Purification Kit (Qiagen) and sent to the Australian Genome Research Facility (AGRF) for sequencing.

Total RNA was isolated from a control larval *A. percula* collected from a 70-litre tank in a closed seawater system at the James Cook University Marine Aquarium Facility using methods described by de Santis et al. (De Santis et al., 2011). Full-length 5′ and 3′ ends were obtained using the GeneRacer^TM^ RACE Kit (Invitrogen) and gene specific primers designed from the partial putative E4 sequence (Table 1; *Ap-5*′end R and *Ap*-3′end F). Amplification was carried out according to GeneRacer^TM^ instructions, including a second “nested” amplification using 1 µL of the first PCR product, GeneRacer^TM^ nested primers and custom designed nested primers, (Table 1; *Ap*5nest and *Ap*3nest). PCR products were then cloned into the pGEM-T Easy Vector system (Promega) and sequenced by AGRF. Sequencing results were verified by aligning each fragment in Geneious Pro 5.0.3 (A. J. Drummond et al., Geneious v5.1, 2010, available from http://www.geneious.com) at their 5′ and 3′ ends to the sequences of their respective GeneRacer^TM^ oligomers and the 374 b.p. putative E4 fragment.

Primers were designed to amplify intron sequences and PCR amplification of each intron was performed using gDNA and Bioline reagents (Table 1). Samples were amplified in C1000 Thermal Cyclers (Bio-Rad) using amplification programs with the following steps: pre-denaturation at 94°C for 2 min; 35 cycles of denaturation at 94°C for 30 sec, annealing at temperatures listed in Table 1 for 30 sec, and extension at 72°C at times listed in Table 1; and, finally, a 10 min extension at 72°C.

Five bands from the Intron 1 amplification were gel extracted using the QIAquick Gel Extraction Kit (Qiagen) and re-amplified. PCR products from each intron were purified using the QIAquick PCR purification kit (Qiagen) and sent to AGRF for sequencing. Sequences obtained were verified by alignment to the flanking exon sequences, in which the primers were designed.

### Sequence and phylogenetic analysis

Nucleotide sequences for the various exons of the *Ap-otx2* were compiled using Geneious Pro 5.0.3 (A. J. Drummond et al., Geneious v5.1, 2010, available from http://www.geneious.com). The coding sequence was translated into amino acids (AAs) using Geneious Pro 5.0.3 (A. J. Drummond et al.) and aligned to forty-six AA sequences from the National Centre for Biotechnology Information (NCBI) using the MUSCLE algorithm (Biomatters) with default settings in Geneious Pro 5.0.3 (A. J. Drummond et al.). Sequences from NCBI that were only partial or extended well beyond the 260 AA Ap-OTX2 sequence at either the N- or C-terminus were excluded. ProtTest was used to determine the best protein model (Abascal et al., 2005) and *MEGA* version 5 (Tamura et al., 2011) was used to evaluate evolutionary relationships by generating maximum likelihood (ML), maximum parsimony (MP) and neighbour joining (NJ) trees, with no outgroup. MP and NJ were analysed with 1000 bootstrap replicates. Fifty percent majority rule consensus support values from all bootstrap replicate analyses were presented on the consensus ML 100 bootstrap replicate tree.

### Anemonefish odour treatments

*A. percula* larvae were exposed to behaviourally relevant settlement odours to examine patterns of *Ap-otx2* expression in the olfactory organ. Larval *A. percula* were obtained from adult breeding pairs maintained at James Cook University's Marine and Aquaculture Research Facility Unit, and were reared using standard methods described by Dixson et al. (Dixson et al., 2008). Groups of newly hatched larvae were split equally into three 70-litre rearing tubs containing artificial seawater (Red Sea Brand). To test effects of a known olfactory settlement cue, 10 grams of leaves from a common coastal rainforest tree, *Xanthostemon chrysanthus*: Myrtaceae, were added to one of the rearing tubs in a nylon bag and either (1) changed daily or (2) added only for the first 24 hours post-hatching. In the second rearing tub, 10 grams of leaves from the swamp tree, *Melaleuca nervosa*, were added daily as the pungent oils released by the leaves are known to elicit an avoidance response by settling anemonefish (Munday et al., 2009). Tree leaf odour was evaluated and not anemone odour or artificial PEA as the *Xanthostemon* and *Melaleuca* leaf cues have been shown to elicit strong positive and repulsive behavioural responses, respectively, in both wild and lab-reared settlement-stage larvae (Munday et al., 2009). Recent experiments demonstrate that positive and negative behavioural imprinting can occur when larvae are exposed to tropical plant odours early in their larval stage (D.L.D., G.P. Jones, P.L.M., M.S. Pratchett, S. Planes and S.R. Thorrold, unpublished data). Therefore, exposing the larvae to leaf cues provides a more powerful test for gene expression than would be possible if using anemone cues alone, which are generally favoured by anemonefish (Elliott et al., 1995; Arvedlund et al., 1999). While we cannot exclude a role for gustation or other chemosenses, the *Xanthostemon* and *Melaleuca* leaf cues are involved in navigation and habitat selection (Munday et al., 2009) and are thus most likely detected by the larval fishes well developed olfactory system. The final rearing tub contained no added settlement-specific olfactory cues in order for the anemonefish to serve as controls. Newly hatched larvae and 11 day post-hatching larvae (the approximate age at which *A. percula* settle; Almany et al., 2007) from control and odour-treatment tanks were snap frozen in liquid nitrogen and stored at −80°C until processing.

### *In-situ* hybridisation

*In-situ* hybridisation was used to test if *Ap-otx2* is expressed in the olfactory area of larval *A. percula.* A 761 b.p. *Ap-otx2* probe for *in-situ* hybridisation was designed from the above mentioned *Ap-otx2* sequence. *A. percula* cDNA was PCR amplified with forward (5′-UCUUUUACAUCCGUCAGUGGGC-3′) and reverse (5′-CCAAGCAAUCGGCAUUGAAGTT-3′) primers designed using Primer3 (Rozen and Skaletsky, 2000) and cloned into the pCR 4-TOPO vector (Invitrogen). Digoxigenin-labelled sense and antisense mRNA probes were transcribed using T3 and T7 RNA polymerases, respectively, according to manufacturer instructions (Roche).

Snap-frozen newly hatched and 11-day-old larvae were thawed at 4°C overnight in 4% paraformaldehyde phosphate-buffered saline. The head was dissected from the body and the eyes were removed. Whole-mount *in-situ* hybridisation was then performed on the dissected, eyeless head as described by Ghosh et al. (Ghosh et al., 2009). Whole mounts were stained with NBT/BCIP (Roche) at 4°C and imaged in PBS using a Leica M165FC stereo dissection microscope. All control and odour treated larvae were processed together in the same solutions for the same amount of time and were compared side-by-side. Significant differences in *Ap-otx2* transcript abundance was determined by comparing olfactory area optical density from each treatment using Image J (NIH) and ANOVA for each replicate image. Fisher's LSD *post hoc* tests were performed as necessary. All statistics were completed with Statistica 8.0 (StatSoft).

## Results

### *Ap-otx2* sequence

**Fig. 1.**
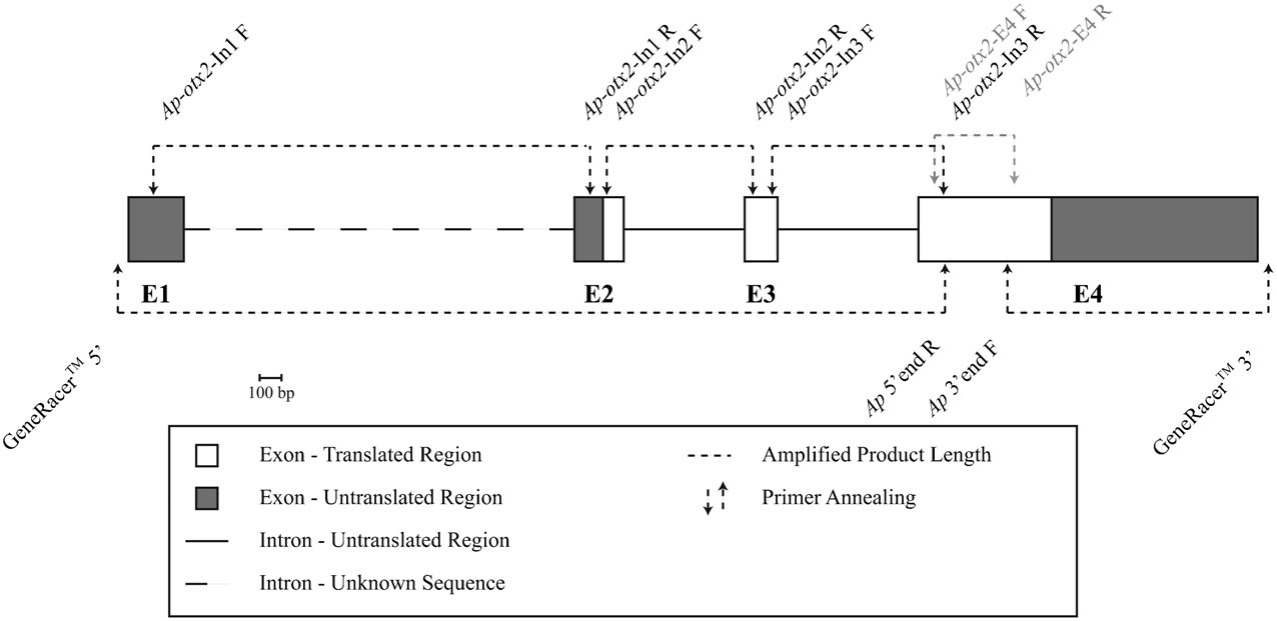
Schematic of the *otx2* gene in *Amphiprion percula*. Each rectangle represents an exon (E1–E4) and each solid line represents an intron. The dashed line represents intron 1 and is depicted as the same size as intron 1 in *otx2* of *Danio rerio*. Grey rectangles correspond to untranslated regions within the exons, while white rectangles correspond to coding regions. Gray dotted arrows indicate the location where the first primers annealed for the initial amplification of the 374 base pair (b.p.) region. The black dotted arrows pointing upwards are the primer annealing points when using the GeneRacer (Invitrogen) kit to amplify the 5′ and 3′ ends and the arrows pointing downwards are where the primers annealed to amplify each intron. Horizontal dotted lines represent amplicon lengths. Scale indicates distance for 100 b.p. Note that nested primers indicated in Table 1 are not included in this figure.

**Fig. 2.**
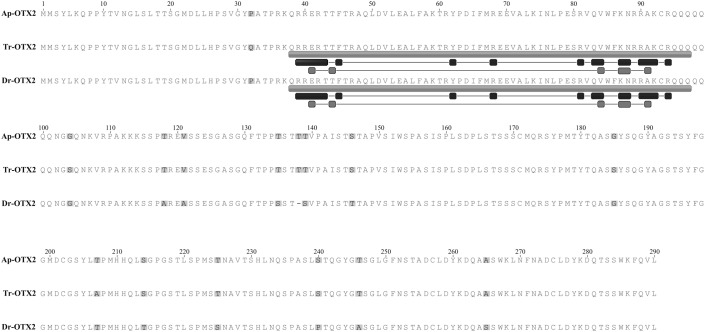
Amino acid sequence alignments for OTX2 from anemonefish, *Amphiprion percula* (Ap-OTX2); pufferfish, *Takifugu rubripes* (Tr-OTX2); and zebrafish, *Danio rerio* (Dr-OTX2). Annotations for Tr-OTX2 and Dr-OTX2 obtained from the GenBank Public Database are indicated below the sequence: the homeodomain (medium-grey rectangle; residues 38 to 97), DNA binding residues (black squares; residues 39–43, 45, 62, 68, 81, 83–84, 87–88, 90–92 and 94) and specific DNA base contact residues (dark-grey squares; residues 41, 44, 84, 88–89 and 91). Disagreements in amino acids from each sequence are highlighted in light-grey.

Based on the *Dr-otx2* sequence, a 374 b.p. region on the putative fourth exon was targeted in *A. percula* as a starting point to determine the full *Ap-otx2* sequence. All four individuals analysed produced identical 374 b.p. sequences. The GeneRacer kit was then used to generate two fragments of 764 b.p. and 1171 b.p. representing the 5′ and 3′ ends, respectively. The two fragments were aligned to *Dr-otx2* exons to determine exon–intron boundaries and the putative four exons are 258 b.p., 231 b.p., 154 b.p. and 1585 b.p. in length, respectively. The nucleotide sequence has a predicted start codon (ATG) at base 134 in E2 and a putative stop codon (TGA) at base 620 in E4 (Fig. 1). Based on this open reading frame, E1 is fully non-coding and E2 has both non-coding (134 b.p.) and coding (97 b.p.) regions, similar to E4 (622 b.p. and 963 b.p., respectively). Overall, the putative coding region is 873 b.p. long and translates to 291 AAs, similar to *T. rubripes* OTX2 (Tr-OTX2) but one AA longer than Dr-OTX2 (Fig. 2).

The putative Ap-OTX2 AA sequence (GenBank accession number JN831750) shows a number of shared motifs with OTX2 AA sequences from other species, including those from the paralogues, OTX1, OTX5, OTX and CRX. The homeodomain, a 60 AA region that binds to DNA (Kelley et al., 2000), is highly conserved and is identical in sequence when compared to Dr-OTX2, a number of cichlids (*Tropheus duboisi*, *Astatoreochromis alluaudi*, *Haplochromis brownae*), several other species of fish (*T. rubripes*, *Oryzias latipes*, *Oryzias melastigma*, *Carassius auratus*), two species of frog (*Xenopus (Silurana) tropicalis*, *Eleutherodactylus coqui*), chicken (*Gallus gallus)*, and a variety of mammals (*Mus musculus*, *Macaca mulatta*, *Rattus norvegicus*, *Homo sapiens*). Interestingly, it is also identical to the OTX homeodomain of the sea squirt, *Herdmania curvata*. Of thirty-two other OTX homeodomain sequences, Ap-OTX2 homeodomain had sequence identities ranging from 72–98%. Eight OTX1 homeodomain sequences matched from 90–98%, ten OTX5 from 95–98%, ten CRX from 87–98% and the other five OTX2 sequences matched from 88–98%. When comparing to annotations from other OTX2 sequences obtained through NCBI (supplementary material Table S1), the Ap-OTX2 homeodomain shares the seventeen AAs responsible for DNA binding and the six that are specific DNA base contacts (Fig. 2).

While introns 2 and 3 produced sequences of 565 and 658 b.p., respectively (Fig. 1), it was not possible to acquire the sequence for intron 1 in this study, despite multiple PCR optimisations and sequencing of the generated PCR products. When a BlastN search was performed on intron 2 and 3, there were no matches to other *otx* sequences. Similarly, the untranslated E1 does not align to any other *otx* sequence. The 133 b.p. untranslated portion of E2, however, has a single match, with an 85% sequence identity to *otx2* from the pufferfish, *T. rubripes* (*Tr-otx2*). In contrast, the 963 b.p. untranslated region of E4 has a region of approximately 155 b.p. that aligns to *otx2* sequences from *G. gallus* (88%; *Gg-otx2*), *Cynops pyrrhogaster* (85%; *Cp-otx2*), *M. mulatta* (85%; *Ma-otx2*), *H. sapiens* (85%; *Hs-otx2*), *X. (Silurana) tropicalis* (83%; *Xst-otx2*), *X. laevis* (82%; *Xl-otx2*) and *R. norvegicus* (81%; *Rn-otx2*). *Tr-otx2* aligns to a much greater portion of the untranslated E4 (72%), with two major sections aligning at 71% and 82% identity, with the 155 b.p. included in the latter section. *Dr-otx2* also aligns to the *Ap-otx2* 155 b.p. region with an additional 70 bases at the 3′ end with an 88% match.

### Phylogenetic analyses

**Fig. 3.**
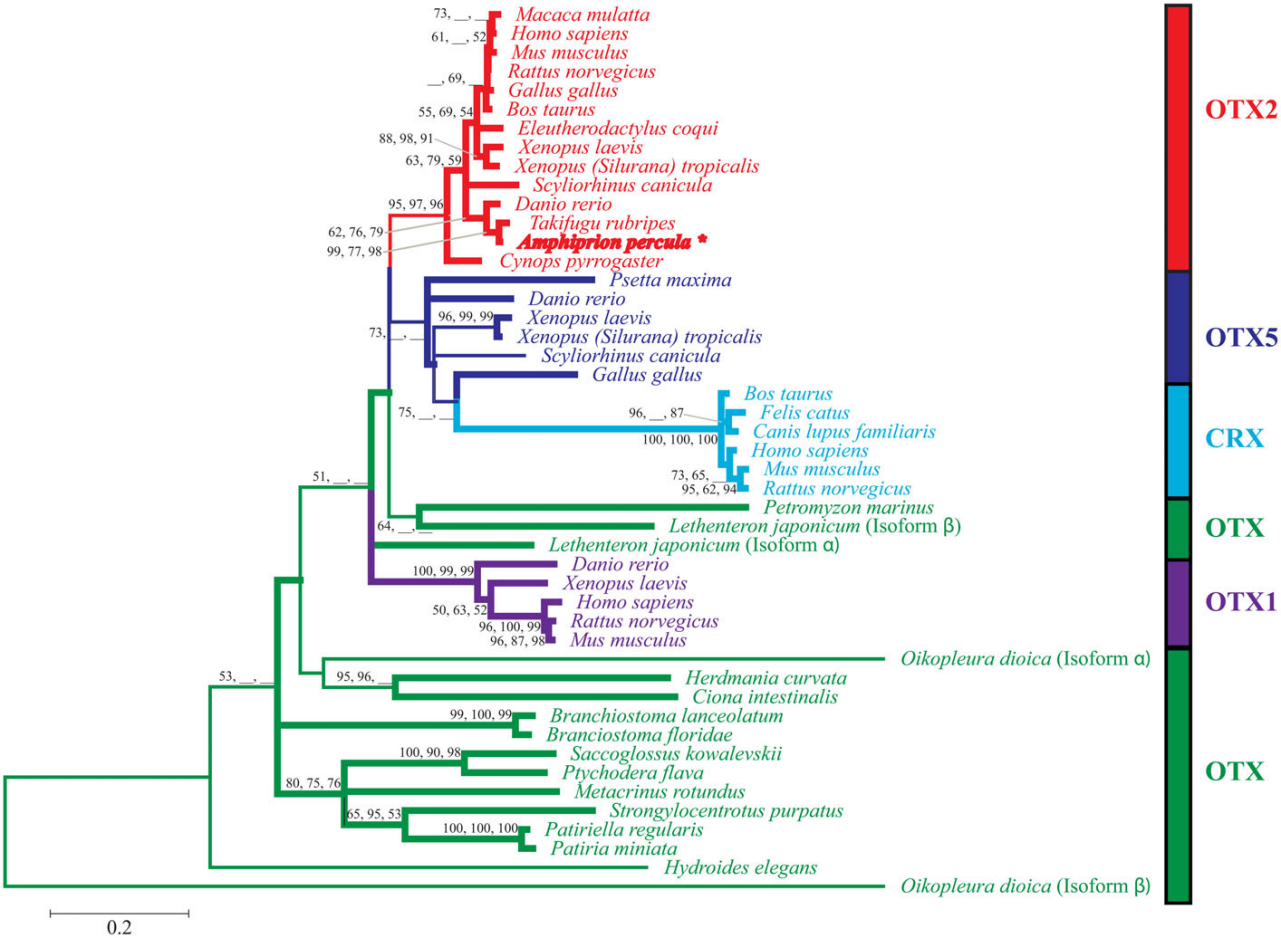
Maximum likelihood (ML) tree of forty-seven OTX amino acid sequences. Values on inter nodes indicate bootstrap support values obtained from 100 ML, 1000 NJ and 1000 MP bootstrap replicates. A lack of support is indicated by ( __ ). The different colours correspond to the different OTX paralogues: OTX  =  green, OTX1  =  purple, OTX5  =  dark blue, CRX  =  light blue and OTX2  =  red. These colours also correspond to the vertical rectangles, which serve to further differentiate the paralogues. The asterisked and bolded selection identifies the species from this study: the anemonefish, *Amphiprion percula*.

From the alignment of 684 AA positions, 287 were parsimony informative, 458 were variable and 143 were singletons. Phylogenetic analyses of aligned AA sequences indicate the separation into five classes: OTX2 (*n* = 14), OTX5 (*n* = 6), CRX (*n* = 6), OTX1 (*n* = 5) and OTX (*n* = 16) (Fig. 3). However, of the forty-seven sequences, OTX from the Sea Lamprey, *Petromyzon marinus* (Pm-OTX), and the two isoforms of the Arctic Lamprey, *Lethenteron japonicum* (Lj-OTXa and Lj-OTXb) do not fall within the OTX class. The analyses also indicate that the deeper nodes have either very little or no support whereas the majority of the shallow nodes are well resolved with mostly strong statistical support.

The sequence obtained in this study is most closely related to other OTX2 sequences, and this clade has the strongest support as a distinct lineage at ≥95%. Therefore, the strong support in this phylogenetic analysis has confirmed that the sequence amplified and characterised in this study is Ap-OTX2 and not one of the other OTX paralogues. Ap-OTX2 is most similar in these analyses to Tr-OTX2 with ≥77% support. In OTX2 and OTX1 lineages, it is clear that fishes are grouped together, followed by frogs and then birds and mammals. The OTX2 sequence for the Japanese Fire Belly Newt, Cp-OTX2, is a sister sequence to the other OTX2 sequences. OTX5 shows a similar arrangement to OTX1 and OTX2, with fish leading to frog and bird; however, no mammal sequences are represented. The OTX class is represented by invertebrate, urochordate and cephalocordate animals, with the exception of the vertebrate Lamprey's (Pm-OTX, Lj-OTXa and Lj-OTXb) which are not found within this group.

### *In-situ* hybridisation

**Fig. 4.**
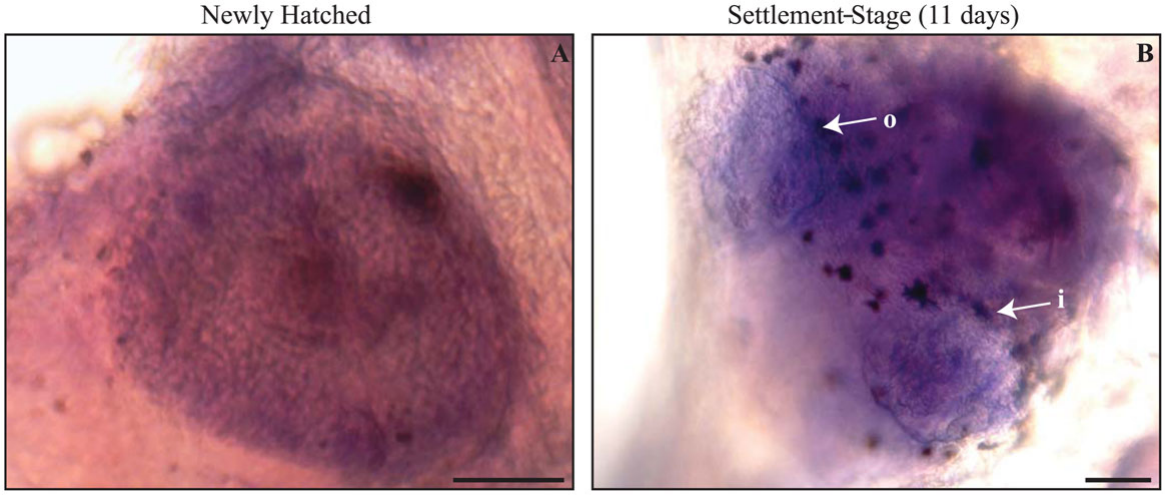
*Ap-otx2 in-situ* hybridisation of the olfactory area in *Amphiprion percula*. Dark purple areas represent *Ap-otx2* mRNA transcript expression in the olfactory placode of a newly hatched larva (**A**) and the olfactory epithelium of settlement-stage larva exposed to *Xanthostemon* leaves (**B**). Circular inlet (i) and outlet (o) nostrils are indicated (B). Dark brown patches are pigment cells. Scale bars: 50 µm.

Both newly hatched and settlement-stage larvae exhibited positive staining for mRNA transcripts of *Ap-otx2* in the olfactory area (Fig. 4). In newly hatched larvae, *Ap-otx2* mRNA transcripts were present throughout the olfactory placode (Fig. 4A). The olfactory placode of *A. percula* was in the same shape and location to that of *Amphiprion melanopus* at a similar developmental stage (Arvedlund et al., 2000b). In settlement-stage larvae, the *Ap-otx2* mRNA transcript was detected throughout the olfactory area, giving a distinct cup shape with defined edges (Fig. 4B). Settlement-stage fish had developed an inlet (anterior) and outlet (posterior) nostril on either side of the head, between the mouth and eye (Fig. 4B). The antisense probe performed in parallel with the control sense probe demonstrated the presence of specific hybridisation in the olfactory area (supplementary material Fig. S1).

**Fig. 5.**
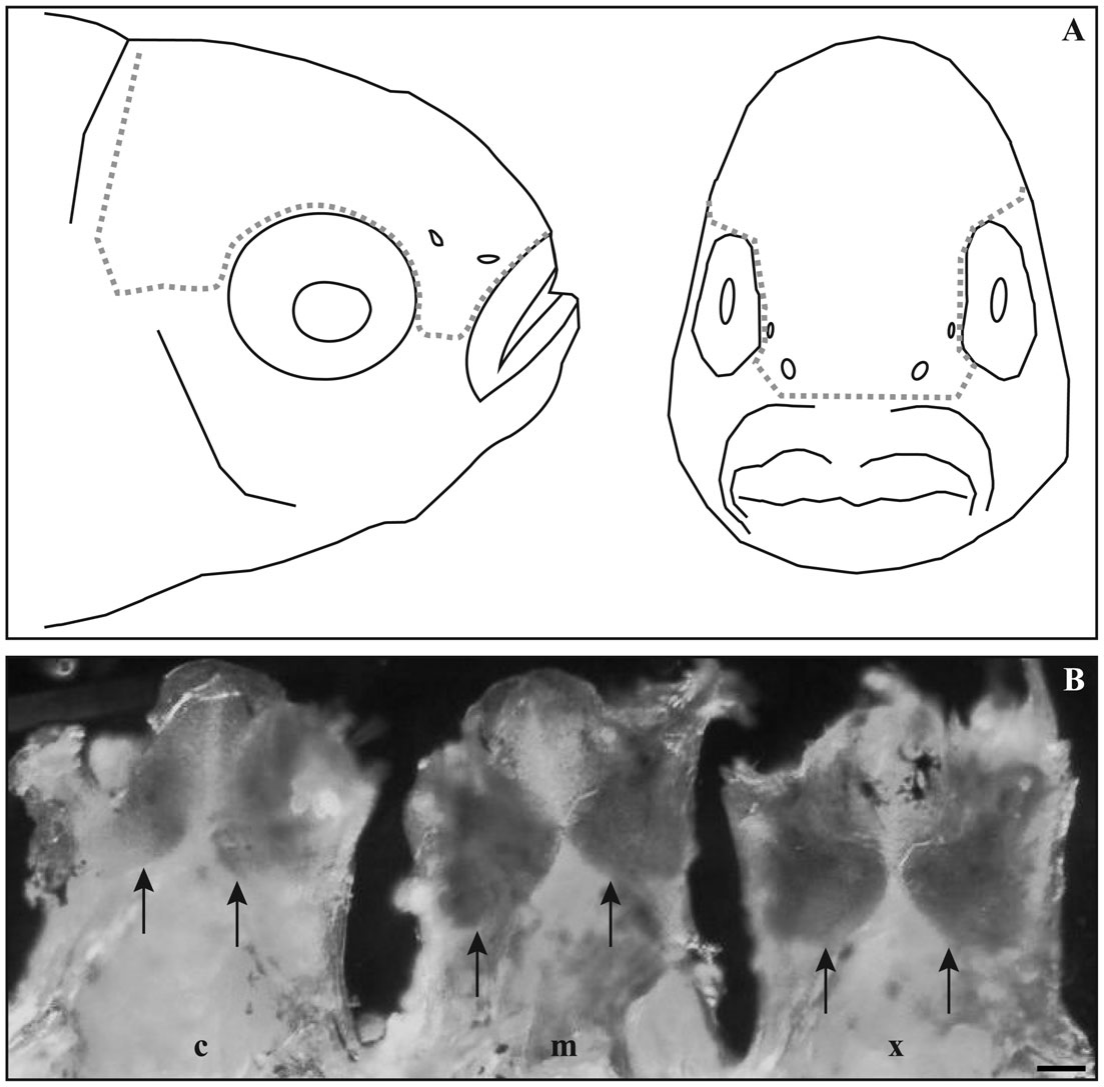
Effect of daily settlement cue odour on *Ap-otx2* expression in the olfactory area after *in-situ* hybridisation. (**A**) Diagram indicating approximate location of tissue dissected from the dorsal section of larval heads (hashed lines). (**B**) Ventral view of three settlement-stage *Amphiprion percula* heads for control (c), *Melaleuca-*(m) and *Xanthostemon*-(x) leaf odour exposed larvae, dissected along the anterior–posterior axis with the anterior-most region (upper jaw) located at the top of the figure. *Ap-otx2* transcripts are restricted to the olfactory area (arrows) and all other black patches represent pigment cells. Scale bar: 170 µm.

The effect of daily exposure to known behavioural settlement cues (attractive and repulsive) on *Ap-otx2* expression in 11-day-old larvae was also evaluated. To better visualise the detection of *Ap-otx2* mRNA transcripts in the olfactory chambers, the ventral portion of the head was removed (Fig. 5A). Evaluation of optical density showed significantly lower levels of *Ap-otx2* transcript staining in the control settlement-stage larvae compared to both the attractive (*Xanthostemon*) and repulsive (*Melaleuca*) leaf odour-exposed *A. percula* for each replicate image (Fig. 5B; *n* = 4; replicates not shown). There was, however, no significant difference in *Ap-otx2* transcript staining between the two settlement-odour treatments.

**Fig. 6.**
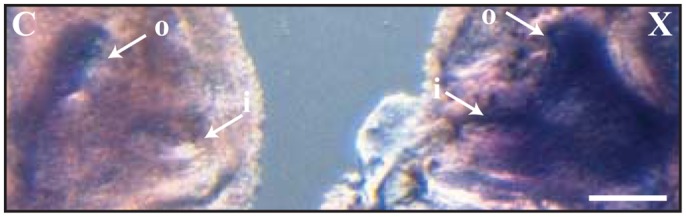
Effect on 11-day old *Amphiprion percula Ap-otx2* transcript abundance after settlement odour exposure for 1-day post-hatching. Lateral view after *in-situ* hybridisation of eyeless heads for control (C) and *Xanthostemon*-(X) leaf odour exposed larvae. Note that control fish were not exposed to settlement cues at any stage. *Ap-otx2* transcripts are located in the olfactory area with dorso- and ventral-boundaries denoted by arrows and showing greater staining in the *Xanthostemon*-treated fish. Black/brown patches represent pigment cells. Circular inlet (i) and outlet (o) nostrils are indicated. Scale bar: 340 µm.

To evaluate whether *Ap-otx2* plays a potential role in settlement odour memory or imprinting, expression was evaluated in larvae that were exposed only on the first day post-hatching to *Xanthostemon* leaves, and not daily. At settlement, odour exposed larvae showed a significant increase in *Ap-otx2* transcript abundance in the olfactory area compared to no-settlement-odour controls, even when only exposed for the first day post-hatching (Fig. 6; *n* = 3; repeats not shown).

## Discussion

This study elucidated and validated the *otx2* gene in the anemonefish, *A. percula* (*Ap-otx2*). *Ap-otx2* is expressed throughout the olfactory placode of newly hatched *A. percula* and within the olfactory area of settlement-stage larvae. Larvae exposed daily to either positive or negative settlement cues, *Xanthostemon* and *Melaleuca* leaves, respectively, qualitatively show greater *Ap-otx2* transcript staining compared to control larvae that experienced no added settlement cues. In addition, larvae only exposed to *Xanthostemon* on the first day after hatching also showed this elevated transcript abundance compared to control larvae. This suggests that *Ap-otx2* is likely involved in settlement-odour detection and imprinting in *A. percula* and may be suitable for further studies as a marker for olfactory imprinting in anemonefish.

### Ap-OTX2 and its relationship to other OTX sequences

Due to the phylogenetic relationship of the Ap-OTX2 AA sequence with other OTX2 proteins rather than with any of the OTX paralogues, the sequence obtained for *A. percula* in this study can confidently be confirmed as OTX2. Other studies that performed similar OTX phylogenetic analyses (e.g. Germot et al., 2001; Plouhinec et al., 2003; Suda et al., 2009) produced comparable results to those obtained here, albeit with fewer taxa represented. Germot et al. performed phylogenetic analyses on 29 OTX sequences from the various classes, using 148 positions (101 of which are parsimony informative) of aligned OTX AA sequences (Germot et al., 2001). Our study included an additional 18 OTX sequences (47 total), 536 additional AA positions (684 total) and more than twice as many parsimony informative sites (287) and generated similar phylogenetic results. The lack of support at the deeper nodes and strong support at shallow nodes, the grouping into the different OTX classes, and the more diverged CRX sequences from the OTX5 class (indicating orthologous genes) are consistent among this study and previous OTX phylogenetic studies (Germot et al., 2001; Plouhinec et al., 2003; Suda et al., 2009), extending the validity of previous work.

The present study included alignments of the OTX class from a number of invertebrates, urochordates and cephalocordates, which were not included in earlier studies, though Suda et al. included the amphioxus *Branchiostoma floridae* (Suda et al., 2009) and Plouhinec et al. included four ascidian sequences (Plouhinec et al., 2003). Plouhinec et al. noted that the ascidian OTX sequences are a monophyletic group (Plouhinec et al., 2003) and, while Germot et al., Suda et al. and this study did not use an outgroup (Germot et al., 2001; Suda et al., 2009), Plouhinec et al. used these ascidian OTX sequences as outgroups (Plouhinec et al., 2003). With the addition of other invertebrate (echinoderms, acorn worms and a polychaete) and ascidian sequences in the present analysis, the non-vertebrate OTX sequences were identified as more closely related to each other and separated from all other OTX paralogues and OTX vertebrates, though only with a maximum support value of 51%. The only OTX sequences analysed in Germot et al. were from hagfish and lamprey vertebrates (Germot et al., 2001) and, due to lack of support, can be seen as more closely related to each other than the other paralogues. Similarly, in the studies by Suda et al. and Plouhinec et al., the hagfish and lamprey OTX sequences appear in various locations throughout their phylogenetic trees with either little or no support for their placement (Suda et al., 2009; Plouhinec et al., 2003). The results from this study suggest that the vertebrate OTX-class sequences, Pm-OTX, Lj-OTXa and Lj-OTXb, may be monophyletic with the other OTX1, OTX5/CRX and OTX2 classes, and it has been suggested that these three OTX classes were fixed before the gnathostome radiation and after the cyclostomes divergence (Germot et al., 2001; Plouhinec et al., 2003). The addition of more vertebrate OTX-class sequences in future analyses may help elucidate their relationship to the other OTX paralogues.

### Odour-treatment expression analysis

To consider *Ap-otx2* as a candidate gene for testing olfactory imprinting in *A. percula*, it is first necessary to: (1) verify that the gene is associated with the olfactory area, (2) determine if there is differential expression in settlement-cue exposed versus unexposed larvae and (3) detect continued increased expression after initial exposure, even without the daily presence of the odour. The presence of *Ap-otx2* transcripts in the olfactory organs of *A. percula* was confirmed in both newly hatched and settlement-stage larvae. Contrary to the limited and localised (ventral–anterior) expression of *Dr-otx2* in adult and 24- and 72-hours post-fertilisation zebrafish (Harden et al., 2006), *Ap-otx2* was expressed throughout the olfactory area and, qualitatively, within a far greater number of cells. Furthermore, positive (*Xanthostemon*) and repulsive (*Melaleuca*) leaf odour settlement cues added daily to larval tanks caused an upregulation of *Ap-otx2* in the olfactory area of settlement-stage *A. percula*, compared to settlement-stage control larvae lacking this olfactory stimulus. Therefore, *Ap-otx2* is responsive to not only attractive odours for settlement, but also repulsive ones and may be involved in a general settlement-odour imprinting response. Both unexposed control and settlement-stage odour-exposed fish would have experienced other odours in the tanks, such as those produced by conspecifics and food. Combined with the fact that the leaf odours used are known to be behaviourally relevant settlement cues, and that control larvae did not exhibit increased *Ap-otx2* expression despite being exposed to other non-settlement odours in the tanks, the results of this study indicate the continued upregulation of *Ap-otx2* compared to controls is likely the direct result of settlement-specific odours.

As wild *A. percula* are not likely to be exposed daily to settlement odours during their pelagic larval phase, the more ecologically relevant test of odour exposure for only the first 24-h post-hatching demonstrated that the larvae not only continue to respond behaviourally to the odour (Dixson et al., 2011) but that *Ap-otx2* remains upregulated compared to larvae never exposed to settlement odours. Recent studies have demonstrated that larval and juvenile reef fishes have distinct olfactory preferences and that they are able to recognise and respond to chemical odours from the reefs where they ultimately settle (Gerlach et al., 2007; Dixson et al., 2008; Miller-Sims et al., 2011). Anemonefish have a well-developed olfactory placode at hatching (Arvedlund et al., 2000b), which would enable them to detect, and potentially imprint on the odour of natal habitats. Furthermore, behavioural imprinting to preferred habitats has been demonstrated in several species of anemonefish (Arvedlund and Nielsen, 1996; Arvedlund et al., 1999). Therefore, the results of this study indicate that *Ap-otx2* is almost certainly involved in the recognition of olfactory settlement cues in *A. percula* and likely plays a role in imprinting. OTX2 has been linked to opening the critical period for neuron plasticity in the visual cortex of mice (Rebsam and Mason, 2008) and similar processes might be at play in the olfactory system of fishes. For example, the early presence of PEA in juvenile zebrafish that results in an upregulation of *Dr-otx2* (Harden et al., 2006) might be the stage at which olfactory plasticity is opened. From that point forward, *Dr-otx2* is always upregulated when compared to unexposed controls and the zebrafish is likely imprinted to PEA. In this study, the greater *Ap-otx2* transcript abundance in settlement stage larvae that were cue-exposed for only the first 24-hours post-hatching may indicate that *Ap-otx2* plays a similar role in olfactory neuron plasticity in anemonefish. Future experiments evaluating changes in expression of additional genes to settlement odour cues may help elucidate the exact nature of the role *Ap-otx2* plays in olfactory imprinting.

## Supplementary Material

Supplementary Material
