# Supplementary material for: Otx2 expression and implications for olfactory imprinting in the anemonefish, Amphiprion percula
Source: Biol Open. 2013 Jul 17;2(9):907–15. doi: 10.1242/bio.20135496 (PMC3773337; doi:10.1242/bio.20135496)
Supplement: Supplementary Material [file supp_2_9_907__index.html]

Otx2 expression and implications for olfactory imprinting in the anemonefish, Amphiprion percula — Otx2 expression and implications for olfactory imprinting in the anemonefish, Amphiprion percula — Supplementary Material 

# *Otx2* expression and implications for olfactory imprinting in the anemonefish, *Amphiprion percula*

## bio.20135496 Supplementary Material

**Files in this Data Supplement:**

- Supplementary Material - Heather D. Veilleux et al. doi: 10.1242/bio.20135496
