# Supplementary material for: Otx2 expression and implications for olfactory imprinting in the anemonefish, Amphiprion percula
Source: Biol Open. 2013 Jul 17;2(9):907–15. doi: 10.1242/bio.20135496 (PMC3773337; doi:10.1242/bio.20135496)
Supplement: Supplementary Material [file supp_bio.20135496_bio.20135496-s1.pdf]

Biology Open

Supplementary Material

Heather D. Veilleux et al. doi: 10.1242/bio.20135496

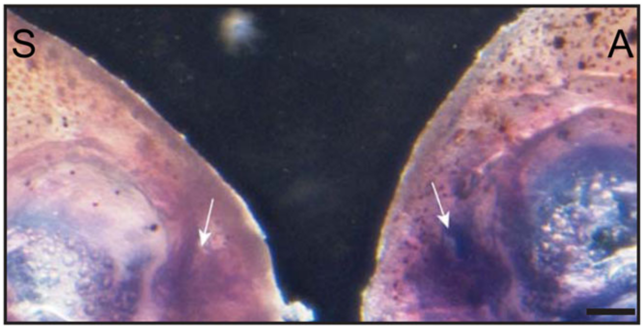

Fig. S1. ***In-situ*** **hybridisation using sense (S) and antisense (A)** ***Ap-otx2*** **probes on 11-day old** ***Amphiprion percula*** **exposed to tropical leaves, showing a lateral view of the heads.** Olfactory area denoted by white arrows. Scale bar: 180 µm.

Biology Open

Table S1. List of species used in the amino acid phylogenetic analyses. The associated OTX paralogue, common name and Genbank accession numbers have been listed.

Otx paralogue	Species	Common name	Genbank nucleotide accession number
CRX	*Bos taurus*	Cattle	NP_776329.1
CRX	*Canis lupus*	Dog	NP_001003049.1
CRX	*Felis catus*	Domestic Cat	ADD10738.1
CRX	*Homo sapiens*	Human	EAW57515.1
CRX	*Mus musculus*	House Mouse	AAH16502.1
CRX	*Rattus norvegicus*	Norway Rat	BAA76666.1
OTX	*Branchiostoma floridae*	Florida Lancelet	AAC00193.1
OTX	*Branchiostoma lanceolatum*	Amphioxus Lancelet	ACE79720.1
OTX	*Ciona intestinalis*	Vase Tunicate	NP_001027662.2
OTX	*Hermania curvata*	Sea Squirt	AAD30505.1
OTX	*Hydroides elegans*	Calcareous Tube Worm	ABK76302.1
OTXa	*Lethenteron japonicum*	Arctic Lamprey	BAA33409.1
OTXb	*Lethenteron japonicum*	Arctic Lamprey	BAA33410.1
OTX	*Metacrinus rotundus*	Stalked Crinoid	ADE59458.1
OTXa	*Oikopluera dioica*	Sea Squirt	AAW88300.1
OTXb	*Oikopluera dioica*	Sea Squirt	AAW82380.1
OTXβa	*Patiria miniata*	Bat Star	AAP32748.1
OTXβ1/2	*Patiriella regularis*	Cushion Seastar	ACX50924.1
OTX	*Petromyzon marinus*	Sea Lamprey	AAC82470.1
OTX	*Ptychodera flava*	Hawaiian Acorn Worm	AAC82470.1
OTX	*Saccoglossus kowalevskii*	Acorn Worm	AAP79293.1
OTXβ	*Strongylocentrotus purpuratus*	Californian Purple Sea Urchin	NP_001027541.1
OTX1	*Danio rerio*	Zebrafish	AAA78900.1
OTX1	*Homo sapiens*	Human	EAW99966.1
OTX1	*Mus musculus*	House Mouse	AAL24809.1
OTX1	*Rattus norvegicus*	Norway Rat	NP_037241.1
OTX1A	*Xenopus laevis*	African Clawed Frog	AAI23256.1
OTX2	*Bos taurus*	Cattle	NP_001180130.1
OTX2	*Cynops pyrrhogaster*	Japanese Fire Belly Newt	BAC53612.1
OTX2	*Danio rerio*	Zebrafish	NP_571326.1
OTX2	*Eleutherodactylus coqui*	Puerto Rican Coqui	AAS49168.1
OTX2	*Gallus gallus*	Chicken	NP_989851.2
OTX2	*Homo sapiens*	Human	NP_068374.1
OTX2	*Macaca mulatta*	Rhesus Monkey	NP_001171116.1
OTX2	*Mus musculus*	House Mouse	AAH29667.1
OTX2	*Rattus norvegicus*	Norway Rat	NP_001094036.1
OTX2	*Scyliorhinus canicula*	Smaller Spotted Catshark	AAP04272.1
OTX2	*Takifugu Pufferfish*	Fugu Pufferfish	AAQ72465.1
OTX2	*Xenopus (Silurana) tropicalis*	Western Clawed Frog	NP_001016177.1
OTX2	*Xenopus laevis*	African Clawed Frog	AAH77357.1
OTX5	*Danio rerio*	Zebrafish	NP_851848.2
OTX5	*Gallus gallus*	Chicken	AB197033.1
OTX5	*Psetta maxima*	Turbot	ABM21525.2
OTX5	*Scyliorhinus canicula*	Smaller Spotted Catshark	AAK85128.1
OTX5	*Xenopus (Silurana) tropicalis*	Western Clawed Frog	Q28EM7.1
OTX5A	*Xenopus laevis*	African Clawed Frog	Q9PVM0.1
